# Development and validation of a cost‐effective virtual reality educational tool to reduce anxiety and improve set‐up accuracy in radiotherapy patients

**DOI:** 10.1002/cam4.5348

**Published:** 2022-10-17

**Authors:** Qianfeng Zhao, Bo Liu, Qiushi Sun, Yiqiang Jin

**Affiliations:** ^1^ Department of Oncology, Xiangyang Central Hospital Affiliated Hospital of Hubei University of Arts and Science Xiangyang China; ^2^ Institute of Oncology Hubei University of Arts and Science Xiangyang China

**Keywords:** patient education, radiotherapy, set‐up accuracy, virtual reality, VR education

## Abstract

**Purpose:**

This study proposes a cost‐effective method for educating radiotherapy patients through an immersive virtual reality (VR) system.

**Methods:**

The VR educational tool comprises VR glasses, a handheld controller, the scientific knowledge of radiotherapy, radiotherapy demonstration, and an audio introduction. To verify its efficacy, 120 radiotherapy patients with tumors were prospectively enrolled and divided into the control group or VR intervention group. After the first treatment, set‐up errors, including three translation errors and three rotation errors, were recorded in six directions. In addition, participants were required to complete a questionnaire before radiotherapy to assess anxiety and understanding degrees. The questionnaire was scored using a five‐point Likert Scale. Finally, Spearman's rank correlation test was used to evaluate set‐up errors and questionnaire scores.

**Results:**

The set‐up errors are significantly reduced in AP, SI, total translation, Roll and total rotation in the intervention group compared with the control group (*p* < 0.05). The scores are higher in the intervention group than in the control group in question 1 (2.1 ± 0.58 vs. 3.3 ± 0.55), question 2 (1.3 ± 0.44 vs. 2.5 ± 0.65), question 4 (2.2 ± 0.65 vs. 3.2 ± 0.82), question 5 (1.8 ± 0.59 vs. 3.1 ± 0.79), and all subscales (5.5 ± 1.2 vs. 8.9 ± 1.3 and 6.4 ± 1.3 vs. 9.2 ± 1.5). The scores of high, moderate, and low correlation are 47 (74%), 15 (23%), and 2 (3%) for the control group and 44 (69%), 17 (26%), and 3 (5%) for the intervention group, respectively.

**Conclusion:**

The VR educational tool can significantly improve comprehension and reduce anxiety. There is a strong correlation between set‐up errors and questionnaire scores. The VR educational tool may help reduce set‐up errors for radiotherapy patients.

## Graphical abstract

This study proposed a virtual reality (VR) tool for educating radiotherapy patients and tested on 120 radiotherapy patients. We found VR educational tool can significantly improve comprehension and reduce anxiety. In addition, this tool is expected to reduce set‐up errors for radiotherapy patients.

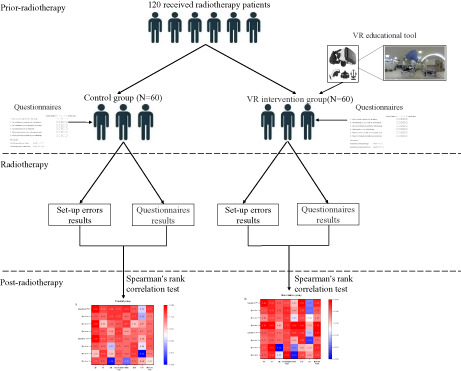

## 1 INTRODUCTION

Radiotherapy is one of the most effective treatments for cancer. More than half of cancer patients undergo radiotherapy as a curative or palliative treatment in the course of their disease.^1^ In the last decade, tumor patients requiring radiotherapy have doubled.^2^ Nevertheless, radiotherapy‐related training remains a bottleneck, especially in low‐ and middle‐income countries that urgently need to expand radiotherapy services.^3^ The problem extends beyond doctors to medical physicists, radiation therapists, nurses, and other health care support roles.

In recent years, an advanced technology, virtual reality (VR), has been applied to the radiotherapy training of physicians.^4^, ^5^, ^6^ However, this tool is rarely used for the radiotherapy education of patients.^7^ Anxiety and stress are common in cancer patients before radiotherapy. Not only is this emotion an unpleasant emotional state, but it may also cause significant psychophysiological difficulties. Even worse, it may lead to muscle tension, less reproducible set‐ups, changes in treatment volumes, and a decrease in overall treatment quality. For example, based on subjective and psychological indicators, a study conducted by Anderson et al.^8^ showed that their study subjects were anxious and troubled when receiving radiotherapy. A report by Stiefel and Razavi^9^ suggested that the feelings of isolation comprise situational anxiety during cancer treatment. As Verres found, patients fear injury when radiation therapy equipment falls on them.^10^ Approximately 40% of patients receiving radiotherapy experienced psychological distress, and 54% experienced expected secondary effects, according to a study conducted by Rahn et al.^11^ Moreover, the use of fixation devices during RT may even lead to claustrophobia in patients.^12^ Halkett et al. concluded that breast cancer patients had higher information needs and anxiety levels before treatment and recommended providing more information to patients in the early stage of radiotherapy.^13^

In most cases, stress and anxiety are caused by patients not knowing what to expect during their treatment. Hence, it is essential to educate patients before radiotherapy to reduce negative emotions. However, patients are usually educated verbally and in writing without any further explanation of what will actually happen in the treatment room.^14^ These problems can easily lead to insufficient delivery of radiotherapy information.

Nowadays, traditional means of delivering education have been supplemented or replaced by video and online materials as well as VR training.^15^, ^16^, ^17^, ^18^ In particular, VR training has been demonstrated to improve comprehension and reduce anxiety related to radiotherapy. The VR system features a stereoscopic computer display (often in the form of goggles) with an immersive 3D environment, whose 6‐degree‐of‐freedom (6‐DoF) spatial tracking can track the movement of the user and controller. Wearers can interact with their virtual environments using this device. The current VR systems are bulky and costly, impeding their wide application in radiotherapy. Thus, an inexpensive and patient‐friendly solution are needed in the daily clinic.

In this research, we develop a cost‐effective tool using immersive VR technology and introduce the tool into the clinic to verify its effectiveness. Using this approach before radiotherapy, we hope to determine if patient education can (a) reduce tension and anxiety, (b) enrich knowledge and positive experiences, and (c) improve patient set‐up accuracy.

## 2 MATERIALS AND METHODS

### 2.1 Patient eligibility criteria and study design

**FIGURE 1:**
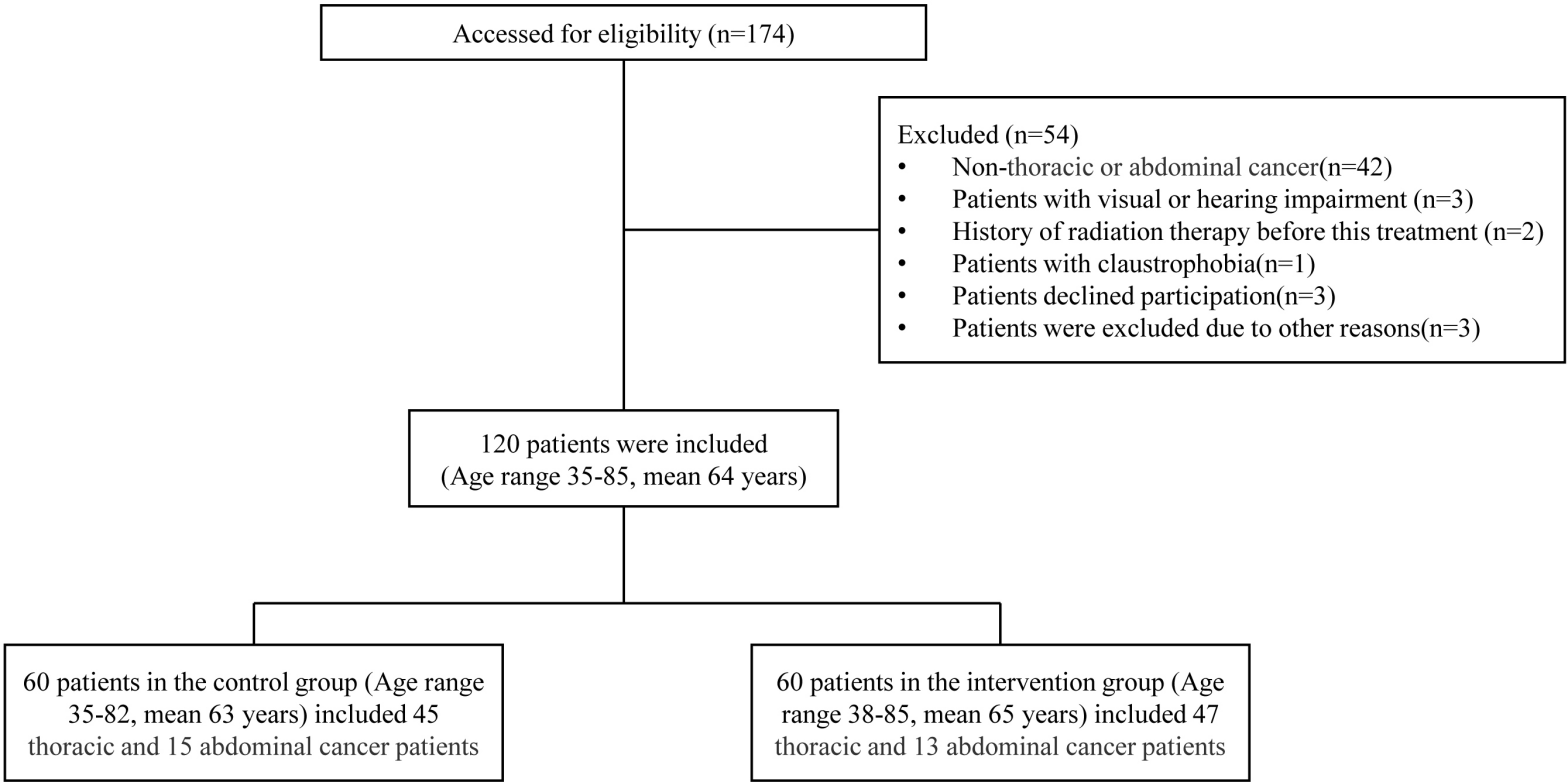
Patient flow chart.

This prospective pilot study of patient education is not a medical experiment but a quality improvement project. All cancer patients provided written and oral informed consent to record their consultations and process their data on audio equipment. All patients treated in the department of radiation oncology at our institution from February 2021 to January 2022 were asked to participate. Patients meeting certain criteria were included in the study: (1) diagnosed with thoracic or abdominal cancer; (2) scheduled to be treated in standard vacuum pad immobilization or thermoplastic mask extended to the shoulders; (3) Chinese as the spoken and written language; (4) age > 18 years; and (5) no apparent impairment in eyesight and hearing. Participants were excluded if they met at least one of the exclusion criteria: (1) history of radiotherapy; (2) known psychiatric condition requiring treatment; and (3) difficulty understanding the nature of the study. The patient flow chart can be seen in Figure 1.

Patients were randomly assigned for standard care (control group) or a VR‐based teaching session (intervention group). The control group was explained verbally by the doctor, and the intervention group combined an additional VR tool. A randomized block design was used to determine the product assignment for clinical investigators. Investigators sequentially received opaque envelopes that contained the assigned products and were prepared by an individual not involved with the clinical aspect of the study. Randomization sequences were created with Stata 9.0 (StataCorp.) statistical software and stratified 1:1 using random blocks.

### 2.2 VR education tool for radiotherapy patients

**FIGURE 2:**
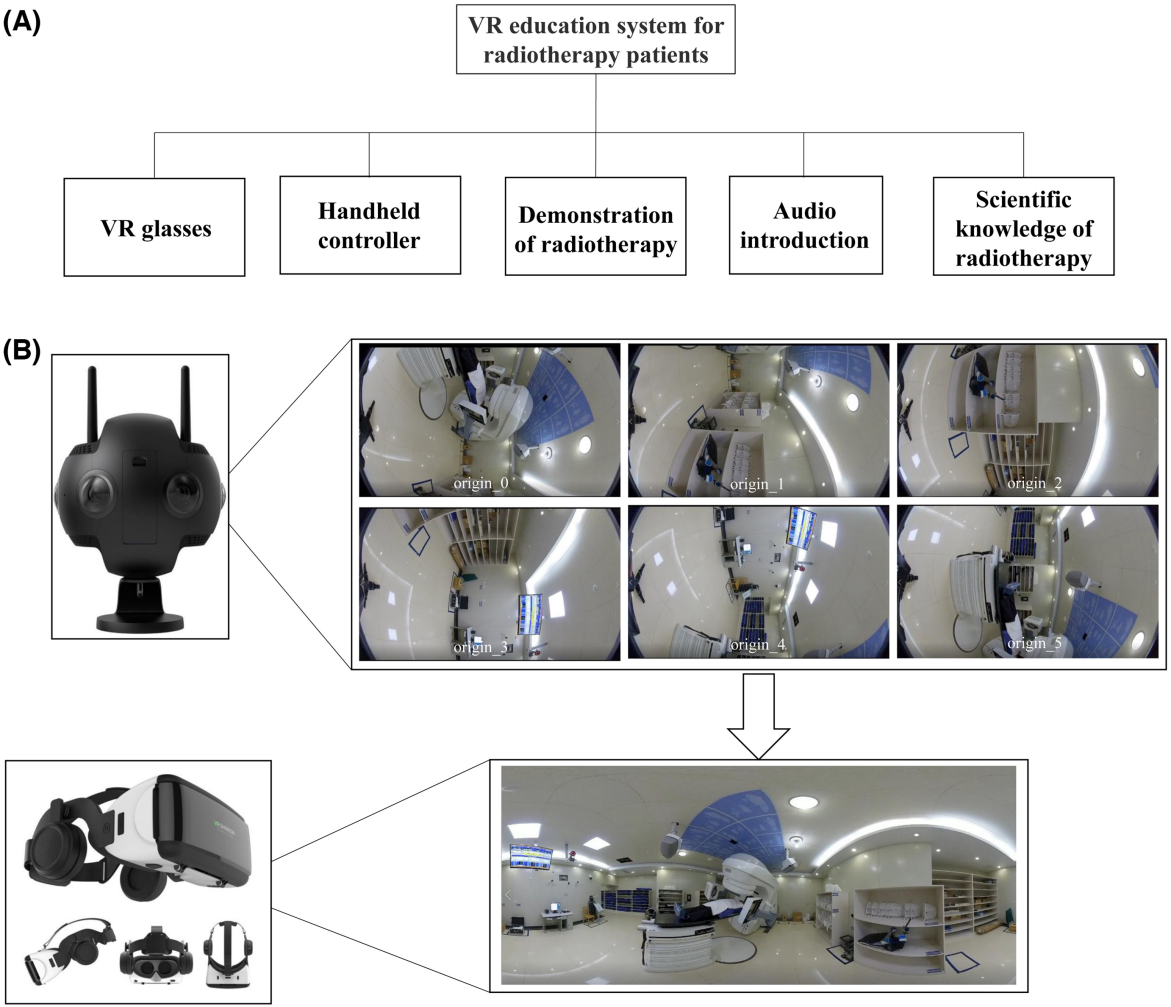
Design of the virtual reality (VR) education tool. (A) The modules of this tool, and (B) the videos in VR glasses.

As shown in Figure 2, the VR tool contains the following components: VR glasses, a handheld controller, scientific knowledge of radiotherapy, a radiotherapy demonstration, and an audio introduction. First, a six‐dimensional camera was used to capture the radiation treatment process of patient volunteers, including entering the radiation treatment room, getting on the treatment couch, moving the body, undergoing the CBCT scan and beam, and getting off the treatment couch after the treatment. Once the images are captured, they are combined into one 3D image and then stored in the VR glasses. The audio introduction is also added to the system.

A 60‐min session of experimenting the VR educational tool was organized 1 week before treatment for all patients in the intervention group. During the experience, they wore VR glasses, turned on the audio system, and watched the stereoscopic radiotherapy process through the handheld controller. In addition, the scientific knowledge of radiotherapy was added to ease the understanding of the concept by the patients. The whole process is repeatable. After the experience, the patients acquired the following knowledge: (1) the environment of the therapy room; (2) the appearance of the radiation therapy equipment; (3) the way to enter and exit the treatment couch; (4) the process of using the fixation devices; (5) the way to adjust breath; (6) the set‐up process; (7) the image‐guided process; (8) the process of position correction; (9) the process of gantry rotation; and (10) some scientific knowledge of radiotherapy.

### 2.3 Radiotherapy

All patients were treated on Varian TureBeam linear accelerator (Varian Medical Systems). Image‐guided radiation therapy was used daily for both groups of patients during their radiotherapy. After the online match and 6‐DoF couch corrections, deviations of patient position can be obtained, including three translational directions (AP: anterior–posterior translation; SI: superior–inferior translation; LR: left–right translation) and three rotational directions (Pitch, Roll and Yaw). Patients with a discrepancy of more than 5 mm between planning CT and daily CBCT were repositioned according to the clinical guidelines. After that, we checked the daily images offline. Any shifts in the target exceeding 4 mm and all repositioned treatments were manually matched to determine if they could be corrected. In cases where this was not possible, it was considered a fraction requiring patient repositioning. The total translational error was defined as $D\left(m\right)=\sqrt{x{\left(m\right)}^{2}+y{\left(m\right)}^{2}+z{\left(m\right)}^{2}}$ for the intrafractional set‐up error, where *D*(*m*) is the magnitude of displacement; *x*(*m*), *y*(*m*), and *z*(*m*) are the displacements in the three orthogonal directions. Similarly, the total rotation error was defined as $D\left(n\right)=\sqrt{p{\left(n\right)}^{2}+r{\left(n\right)}^{2}+y{\left(n\right)}^{2}}$ for the intrafractional set‐up error, where *D*(*n*) is the magnitude of displacement; *p*(*n*), *r*(*n*), and *y*(*n*) are the displacements in the three rotation directions.

### 2.4 Questionnaires and response evaluation criteria

Participants completed a questionnaire before radiotherapy to assess their anxiety and degree of understanding of radiotherapy. Modifications were made to the anxiety scale from the Amsterdam tool (Amsterdam preoperative anxiety and information scale, APAIS) to incorporate an understanding of specific items.^19^ The APAIS has good psychometric properties and is currently the most widely used scale for patient assessment. Nevertheless, it lacks disease‐ and treatment‐specific evaluations. Surgery and radiotherapy are different types of oncology treatment. Therefore, the APAIS cannot be directly used for anxiety scores before radiotherapy. In the modified Amsterdam questionnaire, we changed the term anesthesia to radiotherapy. Additionally, we added an assessment of the understanding of the radiotherapy set‐up process.

**FIGURE 3:**
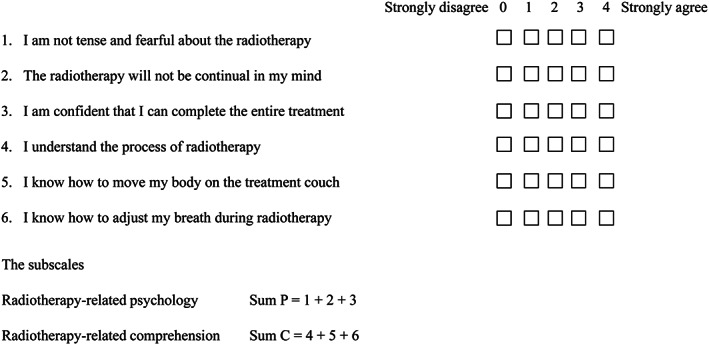
Questionnaires about the degree of anxiety and understanding of radiotherapy.

The six questions contained in the questionnaire were rated by a Likert scale (Figure 3). We defined question 1, question 2, …, as the first question, the second question, …, of the questionnaire, respectively. In this survey, five response degrees were “strongly agree,” “agree,” “undecided,” “disagree,” and “strongly disagree,” with values ranging from 0 (strongly disagree) to 4 (strongly agree). Finally, to quantitatively analyze the difference between the two groups, responses were coded, and statistics were collected. Higher scores indicate higher agreement with the statement (psychology section) or higher patient perception of knowledge (comprehension section).

### 2.5 Statistical analysis

Data (mean and standard deviation) were analyzed descriptively using frequencies and percentages. When the data were normally distributed, a paired student's *t*‐test was used for both groups. As an alternative to paired student's *t*‐test, the Mann–Whitney *U* test was used in cases where the data did not fit the normal distribution. In addition, a Spearman's rank correlation test was used to evaluate set‐up errors and questionnaire scores. The correlation coefficient |*r*| ≥ 0.7, 0.4 ≤ |*r*| < 0.7 and |*r*| ≤ 0.3 indicates the high correlation, moderate correlation, and low correlation, respectively. All tests were performed at the standard level of significance, *p* < 0.05. Data analysis was performed using IBM SPSS for Windows, Version 22 (IBM Corporation).

## 3 RESULTS

### 3.1 Demographics

A total of 120 patients (60 thoracic tumor patients and 60 abdominal tumor patients) treated with radiotherapy from Xiangyang Central Hospital were enrolled in this study. Table 1 lists the baseline characteristics of patients. No significant differences were found among patients in the two groups in any of the variables recorded (Table 1), including gender, age, tumor stage, KPS score, and education level.

**TABLE 1:** Demographic characteristics of patients

Demographic variable	Control group (*N* = 60)	Intervention group (*N* = 60)
Gender		
Male	39 (65%)	35 (58%)
Female	21 (35%)	25 (42%)
Age ranges (years)		
<45	5 (8%)	3 (5%)
45–60	13 (22%)	17 (28%)
>60	42 (70%)	40 (67%)
Tumor sites		
Thoracic tumor	45 (75%)	47 (78%)
Abdominal tumor	15 (25%)	13 (22%)
Tumor stages		
I	0 (0%)	1 (2%)
II	6 (10%)	8 (13%)
III	41 (68%)	40 (67%)
IV	13 (22%)	11 (18%)
Level of education		
No	2 (3%)	1 (2%)
Primary	11 (18%)	8 (13%)
Middle	16 (27%)	19 (32%)
High	22 (37%)	21 (35%)
College	9 (15%)	11 (18%)
Language		
Chinese	60 (100%)	60 (100%)
Others	0 (0%)	0 (0%)
KPS score		
<60	16 (27%)	14 (23%)
60–90	38 (63%)	42 (70%)
>90	6 (10%)	4 (7%)

### 3.2 Set‐up errors for the first treatment session

Table 2 shows the set‐up errors of the online match for each patient's first treatment session. There is a significant reduction in translation and rotation set‐up errors in the intervention group compared with the control group. No differences are found in LR and Yar directions in the two groups.

**TABLE 2:** The set‐up errors of the online match for each patient's first treatment session

Setup errors	Control group (*N* = 60)	Intervention group (*N* = 60)	*p* value
Translational (mm)			
AP	1.5 ± 1.2	1.1 ± 0.83	<0.05
SI	2.1 ± 1.5	1.3 ± 0.81	<0.05
LR	1.4 ± 1.2	1.1 ± 0.82	0.19
Total	2.7 ± 1.9	1.9 ± 1.3	<0.05
Rotation (°)			
Pitch	0.62 ± 0.33	0.55 ± 0.32	0.16
Roll	0.85 ± 0.56	0.65 ± 0.43	<0.05
Yaw	0.52 ± 0.41	0.52 ± 0.38	0.23
Total	1.1 ± 0.62	0.99 ± 0.76	<0.05

Abbreviations: AP, anterior–posterior translation; LR, left–right translation; SI, superior–inferior translation.

### 3.3 Questionnaires

All 120 questionnaires were returned without missing items. As shown in Table 3, significant differences exist between the two groups. The scores are higher in the intervention group than in the control group in question 1 (2.1 ± 0.58 vs. 3.3 ± 0.55), question 2 (1.3 ± 0.44 vs. 2.5 ± 0.65), question 4 (2.2 ± 0.65 vs. 3.2 ± 0.82), question 5 (1.8 ± 0.59 vs. 3.1 ± 0.79), and all subscales (5.5 ± 1.2 vs. 8.9 ± 1.3 and 6.4 ± 1.3 vs. 9.2 ± 1.5).

**TABLE 3:** The results of the questionnaires

Question	Control group (*N* = 60)	Intervention group (*N* = 60)	*p* value
Psychology			
I am not tense and fearful about the radiotherapy	2.1 ± 0.58	3.3 ± 0.55	<0.05
The radiotherapy will not be continual in my mind	1.3 ± 0.44	2.5 ± 0.65	<0.05
I am confident that I can complete the entire treatment	3.1 ± 0.64	3.2 ± 0.58	0.25
The subscales—radiotherapy‐related psychology	5.5 ± 1.2	8.9 ± 1.3	<0.05
Comprehension			
I understand the process of radiotherapy	2.2 ± 0.65	3.2 ± 0.82	<0.05
I know how to move my body on the treatment couch	1.8 ± 0.59	3.1 ± 0.79	<0.05
I know how to adjust my breath during radiotherapy	2.1 ± 0.63	2.7 ± 0.65	0.13
The subscales—radiotherapy‐related comprehension	6.4 ± 1.3	9.2 ± 1.5	<0.05

### 3.4 Correlation between set‐up errors and questionnaire scores

**FIGURE 4:**
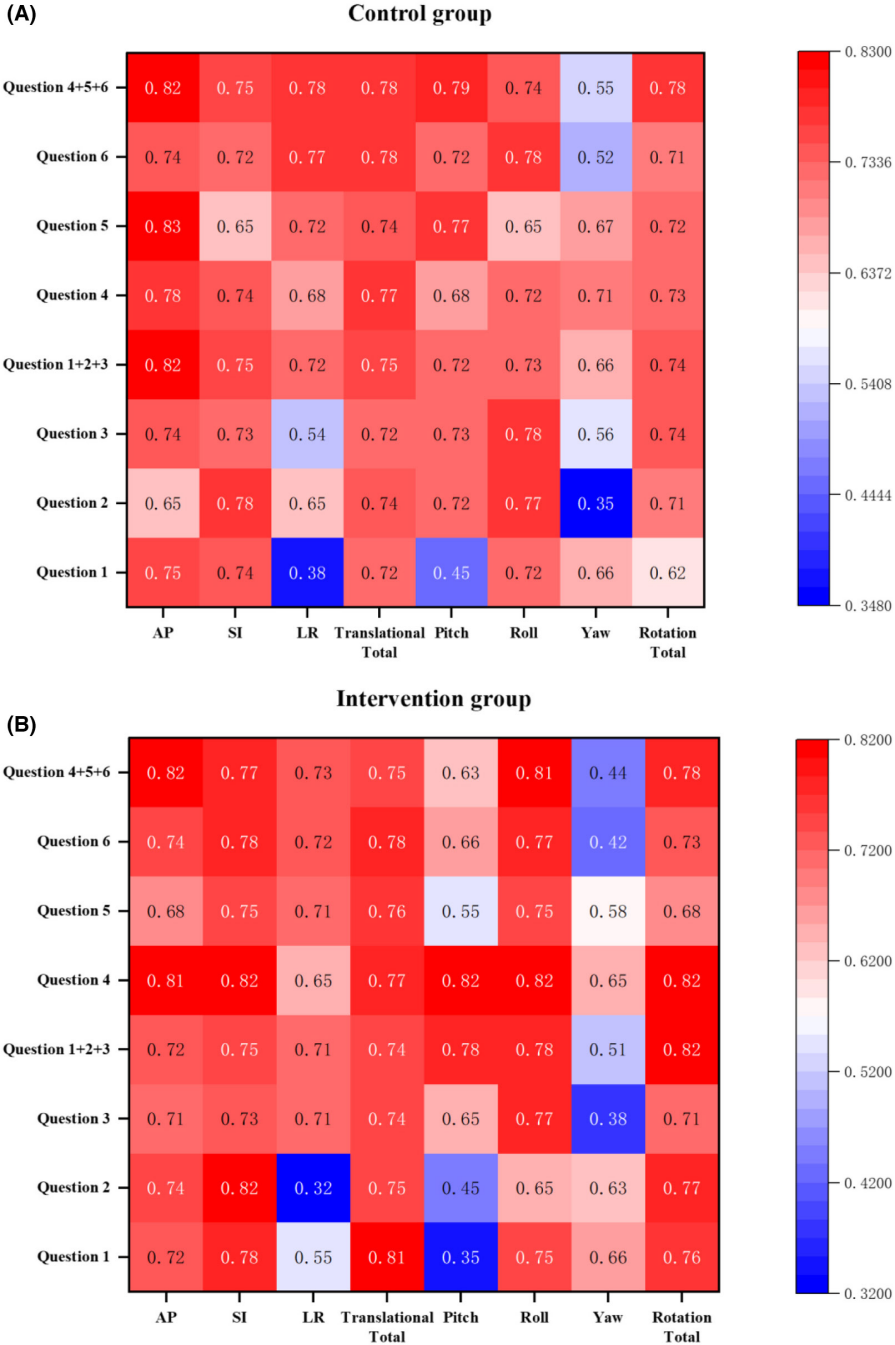
Correlation coefficient (|*r*|) value between set‐up errors and questionnaire scores. (A) is the |r| value in control group. (B) is the |r| value in intervention group.

Figure 4 shows the correlation between set‐up errors and questionnaire scores. The correlation coefficients calculated in this study are all negative. For the control group, the mean of the Spearman correlation coefficient is 0.71. The minimum correlation coefficient is 0.35 in Yaw and question 2, and the maximum is 0.83 in AP and question 5. The scores of high, moderate, and low correlation are 47 (74%), 15 (23%), and 2 (3%), respectively. For the intervention group, the mean of the Spearman correlation coefficient is 0.70. The minimum correlation coefficient is 0.32 in LR and question 2, and the maximum is 0.82 in SI and question 2, The scores of high, moderate, and low correlation are 44 (69%), 17 (26%), and 3 (5%), respectively.

## 4 DISCUSSION

Different from other treatments such as surgery, radiotherapy is a unique form of treatment. Education of radiotherapy patients is essential. A study suggested that visual aids can enhance comprehension and retention of information during cancer education.^15^ Generally speaking, radiation oncologists' oral explanations and 2D videos are the primary sources of information for radiotherapy patients with tumors. However, using the methods above, patients may not fully comprehend how radiotherapy works. For example, one obstacle to radiotherapy is that patients do not have a basic understanding of radiotherapy, and radiation oncologists are unable to explain radiotherapy set‐up knowledge in a simplified, general manner.

As a new method of patient education, VR simplifies the communication between patients and radiation oncologists by materializing the conversation. Virtual environment for radiotherapy training (VERT) is considered the first VR radiotherapy training system that has been used globally since 2007.^20^ It provides a life‐size virtual radiotherapy treatment room with all equipment and a patient on the treatment couch. Research has been conducted across various fields to investigate the benefits of VERT in training radiotherapy students for treatment delivery^12^, ^18^, ^21^ and simulation.^16^

VERT has been applied in medical dosimetry to demonstrate the role of dosimetry in different treatment techniques and assess potential side effects for patients.^16^ Additionally, similar research has been performed on patients. For example, after receiving the VERT information, patients felt that their knowledge increased greatly and recommended this equipment and learning method to other patients receiving radiotherapy.^22^, ^23^ The study by Jimenez et al.^24^ indicated that the comprehensiveness of the education program for patients requires a high priority, with particular acknowledgment of the three‐dimensional visual features of the VERT system. Stewart–Lord^25^ showed that VERT helped to understand the importance of following bowel and bladder preparation protocols. The overall satisfaction and help of VERT were high. Sulé‐Suso et al.^26^ found that 83% of patients had a moderate or high need to better understand how radiology is delivered through 3D imaging. All respondents cited better understanding as a positive outcome.

However, VERT systems can cost millions of dollars. There may be significant limitations to the widespread implementation of VERT due to the additional expenditures incurred by this technology. In this study, we developed an immersive tool for pre‐treatment education of radiotherapy patients. Different from VERT, this tool costs only a few hundred dollars. It is cost‐effective and easy to use, without technical limitations in the tool development process.

We envision that this tool can reduce the tension and anxiety of patients before radiotherapy and improve the set‐up accuracy. The effect of this tool was tested by two (VR group vs. control group) experimental designs. Data analysis confirmed our hypothesis that applying the VR tool could reduce set‐up errors significantly in radiotherapy patients, as suggested in our preliminary study. The results of the questionnaires indicated that this tool reduced patient anxiety before radiotherapy, increased their comprehension, and improved their confidence. In addition, we found a correlation between the magnitude of set‐up errors and the scores of the questionnaires in radiotherapy patients. Thus, reduced anxiety and increased comprehension may contribute to a reduction in patients' set‐up errors.

The study also has some limitations. An unblinded assessment can result in bias, and the population for testing is limited, which may affect the utility of the results. Moreover, because no multi‐center study was conducted, the accuracy of the results may decrease. Furthermore, the questionnaires used in the study were developed according to the APAIS and are not typical or commonly used. Despite that, we are confident that VR technology will become a new paradigm for human‐computer interaction in the future.

## 5 CONCLUSION

Our findings show that the VR educational tool can significantly improve comprehension and reduce anxiety. In addition, this tool is expected to reduce set‐up errors for radiotherapy patients. However, it is important to confirm the benefits of the tool through long‐term follow‐up and multi‐center research. In the future, the module may include customized, patient‐specific features designed to reduce patient anxiety and improve the reproducibility and accuracy of radiotherapy.

## AUTHOR CONTRIBUTIONS

**Qianfeng Zhao:** Formal analysis (equal); methodology (equal). **Bo Liu:** Data curation (equal); formal analysis (equal); investigation (equal); methodology (equal). **Qiushi Sun:** Conceptualization (equal); data curation (equal); methodology (equal). **Yiqiang Jin:** Conceptualization (equal); data curation (equal); funding acquisition (equal); investigation (equal).

## CONFLICT OF INTEREST

None declared.

## ETHICS STATEMENT

This prospective pilot study of patient education is not a medical experiment but a quality improvement project and was approved by the local ethics committee. All cancer patients provided written and oral informed consent to record their consultations and process their data on audio equipment. All procedures performed in studies involving human participants were in accordance with the ethical standards of the institutional and/or national research committee and with the 1964 Helsinki Declaration and its later amendments or comparable ethical standards.

## Data Availability

The data that support the findings of this study are available from the corresponding author upon reasonable request.
